# 血液病患者异基因造血干细胞移植后侵袭性真菌性鼻窦炎的临床特征及危险因素分析

**DOI:** 10.3760/cma.j.cn121090-20231009-00175

**Published:** 2024-01

**Authors:** 海霞 付, 佳佳 李, 圆圆 张, 于谦 孙, 晓冬 莫, 婷婷 韩, 军 孔, 萌 吕, 伟 韩, 欢 陈, 育红 陈, 峰蓉 王, 晨华 闫, 瑶 陈, 景枝 王, 昱 王, 兰平 许, 晓军 黄, 晓辉 张

**Affiliations:** 1 北京大学人民医院，北京大学血液病研究所，国家血液系统疾病临床医学研究中心，造血干细胞移植治疗血液病北京市重点实验室，北京 100044 Peking University People's Hospital, Peking University Institute of Hematology, National Clinical Research Center for Hematologic Disease, Beijing Key Laboratory of Hematopoietic Stem Cell Transplantation, Beijing 100044, China; 2 蚌埠医学院第一附属医院，蚌埠 233003 First affiliated hospital of the Bengbu Medical College, Bengbu 233003, China

**Keywords:** 异基因造血干细胞移植, 真菌性鼻窦炎, 临床特征, 危险因素, Allogeneic hematopoietic stem cell transplantation, Fungal sinusitis, Clinical characteristic, Risk factor

## Abstract

**目的:**

分析血液病患者异基因造血干细胞移植（allo-HSCT）后侵袭性真菌性鼻窦炎（IFR）的临床特征及预后，探索allo-HSCT后发生IFR的危险因素及预后。

**方法:**

纳入2012年1月至2021年12月在北京大学人民医院接受allo-HSCT后并发IFR的19例血液病患者，按照1∶5的比例随机选择95例同期未并发IFR的allo-HSCT患者作为对照组，对两组患者进行回顾性巢式病例对照研究。

**结果:**

19例移植后并发IFR患者中男10例，女9例，中位年龄36（10～59）岁，IFR中位发生时间为移植后68（9～880）d。急性髓系白血病（AML）7例，急性淋巴母细胞白血病（ALL）5例，骨髓增生异常综合征（MDS）2例，慢性髓性白血病（CML）2例，急性混合细胞白血病1例，多发性骨髓瘤1例，T淋巴母细胞样淋巴结瘤1例。确诊13例（68.4％），临床诊断6例（31.6％）。病原菌分布：接合菌6例（31.5％，毛霉菌2例，根霉菌4例），曲霉菌4例（21.1％），念珠菌3株（15.8％）。5例接受两性霉素B+泊沙康唑联合治疗，1例接受伏立康唑+泊沙康唑联合治疗，9例接受伏立康唑单药治疗，4例接受两性霉素B单药治疗。除抗真菌药物治疗外，10例患者接受手术治疗，7例获得疗效。经抗真菌药物及手术治疗后，15例患者（78.9％）患者治疗有效，其中13例（68.4％）达到完全反应，2例（10.5％）为部分反应。多因素分析显示移植前中性粒细胞缺乏（*P*＝0.021）、移植后并发出血性膀胱炎（*P*＝0.012）、血小板延迟植入（*P*＝0.008）、移植物单个核细胞计数较少（*P*＝0.012）是移植后IFR发生的独立危险因素。IFR组、对照组移植后5年OS率分别为（29.00±0.12）％、（91.00±0.03）％（*P*<0.01）。

**结论:**

IFR是血液病患者allo-HSCT后相对少见但对移植预后有严重影响的并发症，全身抗真菌药物联合手术治疗可获得较好的临床疗效。

## Abstract (en)

**Objective:**

To analyze the clinical characteristics and outcomes of patients with invasive fungal sinusitis (invasive fungal rhinosinusitis, IFR) after allogeneic hematopoietic stem cell transplantation (allo-HSCT) and explored the risk factors for IFR after allo-HSCT.

**Methods:**

Nineteen patients with IFR after allo-HSCT at Peking University People's Hospital from January 2012 to December 2021 were selected as the study group, and 95 patients without IFR after allo-HSCT during this period were randomly selected as the control group (1:5 ratio).

**Results:**

Nineteen patients, including 10 males and 9 females, had IFR after allo-HSCT. The median age was 36 (10–59) years. The median IFR onset time was 68 (9–880) days after allo-HSCT. There were seven patients with acute myeloid leukemia, five with acute lymphoblastic leukemia, two with myelodysplastic syndrome, two with chronic myeloid leukemia, one with acute mixed-cell leukemia, one with multiple myeloma, and one with T-lymphoblastic lymph node tumor. There were 13 confirmed cases and 6 clinically diagnosed cases. The responsible fungus was Mucor in two cases, Rhizopus in four, Aspergillus in four, and Candida in three. Five patients received combined treatment comprising amphotericin B and posaconazole, one patient received combined treatment comprising voriconazole and posaconazole, nine patients received voriconazole, and four patients received amphotericin B. In addition to antifungal treatment, 10 patients underwent surgery. After antifungal treatment and surgery, 15 patients achieved a response, including 13 patients with a complete response and 2 patients with a partial response. Multivariate analysis revealed that neutropenia before transplantation（*P*＝0.021）, hemorrhagic cystitis after transplantation（*P*＝0.012）, delayed platelet engraftment（*P*＝0.008）, and lower transplant mononuclear cell count（*P*＝0.012）were independent risk factors for IFR after allo-HSCT. The 5-year overall survival rates in the IFR and control groups after transplantation were 29.00%±0.12% and 91.00%±0.03%, respectively（*P*<0.01）.

**Conclusion:**

Although IFR is rare, it is associated with poor outcomes in patients undergoing allo-HSCT. The combination of antifungal treatment and surgery might be effective.

异基因造血干细胞移植（allo-HSCT）是治疗血液系统恶性肿瘤和部分非恶性疾病的有效方法。侵袭性真菌病（IFD）在allo-HSCT患者中的发生率为3％～23％，病死率高达30％～100％[1]。真菌性鼻窦炎（Fungal sinusitis, FRS）分为非侵袭性和侵袭性（IFR）两种类型。非侵袭性FRS的真菌感染局限于浅表上皮。在IFR中，真菌孢子被吸入鼻腔后，在黏膜下生长，侵犯神经及血管，鼻腔组织出现局部或广泛的缺血性坏死，从而导致受累鼻窦腔中的真菌孢子扩散至周围组织和骨骼[2]，病死率高达50％～80％[3]–[4]。allo-HSCT后合并IFR相关病例报告极少，临床现状不清。因此，本研究对本中心19例allo-HSCT后IFR患者进行回顾性分析，旨在探究allo-HSCT后IFR的临床症状、危险因素、病原体分布、预后和危险因素，以期指导临床诊疗。

## 病例与方法

1. 病例：2012年1月至2021年12月期间在北京大学人民医院接受allo-HSCT的8 798例患者中，19例（0.22％）确诊或临床诊断为IFR。IFR诊断标准参考《血液病/恶性肿瘤患者侵袭性真菌病的诊断标准与治疗原则（第六次修订版）》[5]。按照1∶5的比例随机选择95例同期未合并IFR的allo-HSCT患者作为对照组。

2. 移植措施：移植配型、供者选择、预处理方案、感染及移植物抗宿主病（GVHD）预防措施按我中心常规方案[6]–[10]进行。同胞全相合移植预处理采用以白消安（Bu）、环磷酰胺（Cy）为基础的改良Bu/Cy方案。单倍体移植预处理方案包括：①以Bu、Cy联合兔抗人胸腺细胞免疫球蛋白（rATG）为基础的改良Bu/Cy+ATG方案；②Bu、Cy、氟达拉滨（Flu）联合rATG为基础的Bu/Flu/Cy+ATG方案；③全身放射治疗（TBI）、Cy联合rATG为基础的TBI/Cy+ATG方案。GVHD的预防采用环孢素A加短程甲氨蝶呤联合霉酚酸酯方案。急性GVHD诊断分级参照MAGIC分级标准[11]。

3. IFD的预防策略：对无IFD病史的患者，2020年前，同胞全相合移植后使用氟康唑预防治疗，单倍体移植患者移植后使用伊曲康唑预防治疗；2020年后，所有患者均接受泊沙康唑口服一级预防治疗，持续使用至移植后75 d以上或真菌突破性感染。既往有IFD病史的患者，采用既往有效的抗真菌药物或伏立康唑预防治疗，持续使用至移植后100 d以上或真菌突破性感染。

4. IFR的诊断及疗效评估标准：FRS的诊断与疗效评估均参考《血液病/恶性肿瘤患者侵袭性真菌病的诊断标准与治疗原则（第六次修订版）》[6]。根据患者是否具有宿主因素、临床症状和体征、影像学检查、实验室检查以及微生物学检查将IFR患者分为确诊、临床诊断、拟诊及未确定IFR。IFR治疗完全缓解（CR）：患者在观察期内存活，IFR相关症状和体征、影像学异常全部消失，微生物学检测提示真菌清除；IFR治疗部分缓解（PR）：患者在观察期内存活，IFR相关症状和体征、影像学异常有所改善，微生物学检测提示真菌清除。

5. 观察指标：收集以下临床资料：①患者临床表现；②CT、MRI等影像学检查资料；③鼻窦组织病理学检查资料；④实验室检查：G试验、GM试验、鼻窦组织学培养。

6. 统计学处理：数据分析采用SPSS25.0统计软件进行。分类变量组间比较采用*χ*^2^检验或Fisher精确检验，连续变量组间采用t检验，连续变量值为中位数和极值，分类变量以百分比表示。真菌鼻窦炎的危险因素分别采用单因素和多因素Logistic回归分析分析。纳入患者年龄、性别、移植类型、白细胞计数、中性粒细胞计数、血红蛋白、血小板计数、干细胞来源、预处理方案、移植后是否感染巨细胞病毒（CMV）和EB病毒（EBV）、急性GVHD、移植相关并发症≥3个等作为IFR危险因素进行单因素分析，*P*<0.1的因素纳入多因素分析。总生存（OS）时间定义为从供者干细胞回输后第1天至随访结束或因任何原因死亡的时间。OS采用Kaplan-Meier曲线进行分析，采用Log-rank检验进行比较。所有统计中*P*<0.05（双侧）表示差异有统计学意义。

## 结果

1. IFR患者的临床特征（表1）：19例IFR患者中，男10例，女9例，中位年龄36（10～59）岁；急性髓系白血病（AML）7例，急性淋巴母细胞白血病5例，骨髓增生异常综合征2例，慢性髓性白血病2例，急性混合细胞白血病1例，多发性骨髓瘤1例，T淋巴母细胞样淋巴结瘤1例。17例患者移植前获得完全缓解，2例患者为复发状态；全相合同胞供者移植4例，单倍体移植15例。移植前血常规（中位数）：WBC 3.34（1.03～12.98）×10^9^/L，中性粒细胞计数（ANC）2.37（0.24～5.20）×10^9^/L，HGB 94（67～138）g/L，PLT 112（40～296）×10^9^/L。

**表1:** 19例allo-HSCT后并发侵袭性真菌性鼻窦炎（IFR）患者的临床特征

临床特征	结果
年龄［岁，*M*（范围）］	36（10~59）
性别［例（%）］	
男	10（52.6）
女	9（47.4）
供者［例（%）］	
全相合同胞	4（21.1）
单倍体	15（78.7）
移植前血常规［*M*（范围）］	
WBC（×10^9^/L）	3.34（1.03~12.98）
ANC（×10^9^/L）	2.3（0.2~5.2）
HGB（g/L）	94（67~138）
PLT（×10^9^/L）	112（40~296）
CD34^+^细胞输注量［×10^6^/kg，*M*（范围）］	2.12（0.49~6.39）
MNC输注量［×10^8^/kg，*M*（范围）］	7.47（2.54~16.00）
中性粒细胞植活时间［d，*M*（范围）］	13（11~23）
血小板植活时间［d，*M*（范围）］	17（9~160）
干细胞来源［例（%）］	
外周血	2（10.5）
外周血+骨髓	17（89.5）
预处理方案［例（%）］	
改良Bu/Cy	3（15.8）
改良Bu/Cu+ATG	14（73.7）
Bu/Flu/Cy+ATG	0
Cy+ATG	2（10.5）
移植前化疗次数［例（%）］	
≤3次	9（47.4）
>3次	10（52.6）
糖皮质激素使用［例（%）］	
有	7（36.8）
无	12（63.2）
移植后CMV感染［例（%）］	
有	12（63.2）
无	7（36.8）
急性GVHD［例（%）］	
<Ⅱ度	12（63.2）
Ⅱ~Ⅳ度	7（36.8）
移植后EBV感染［例（%）］	
有	4（21.1）
无	15（78.9）
出血性膀胱炎［例（%）］	
有	3（15.8）
无	16（84.2）
真菌性肺炎［例（%）］	
有	4（21.1）
无	15（78.9）
移植相关并发症［例（%）］	
<3个	7（36.8）
≥3个	12（63.2）

注 AML：急性髓系白血病；ALL：急性淋巴细胞白血病；Bu：白消安；Cy：环磷酰胺；Flu：氟达拉滨；ATG：抗胸腺细胞球蛋白；GVHD：移植物抗宿主病；MNC：单个核细胞；CMV：巨细胞病毒；EBV：EB病毒

在19例IFR患者中，2例（10.5％）患者的移植物为外周血干细胞（PBSC），17例（89.5％）为PBSC+骨髓（BMSC）。单个核细胞（MNC）中位输注量为7.93（2.54～16.00）×10^8^/kg，CD34^+^细胞中位输注量为2.12（0.49～6.39）×10^6^/kg。粒细胞中位植入时间为13（11～23）d，移植后28 d粒细胞植入率为100％。血小板中位植入时间为17（9～160）d，移植后90 d血小板植入率为94.7％。

2. 移植相关合并症：在19例IFR患者中，12例（63.2％）移植后发生CMV感染，4例（21.1％）发生EBV感染；3例（15.8％）发生出血性膀胱炎。EBV感染及出血性膀胱炎均发生在IFR之前；其他移植并发症包括移植相关血栓性微血管病（TA-TMA）1例、移植后淋巴细胞增殖性疾病（PTLD）1例。10例（52.6％）发生急性GVHD，其中3例（15.8％）为Ⅰ度，7例（36.8％）为Ⅱ～Ⅳ度。

3. 真菌预防及突破：IFR组分别1、8、4例接受泊沙康唑、伊曲康唑和氟康唑进行真菌预防，对照组则分别有7、49、13例。对照组中7例接受伊曲康唑预防和2例接受氟康唑预防的患者发生肺部真菌感染，再加上IFR，最终一级预防真菌感染突破率分别为泊沙康唑12.5％（1/8）、伊曲康唑26.3％（15/57）、氟康唑35.3％（6/17）。

4. IFR的临床表现：19例IFR患者中，确诊、临床诊断分别为13例、6例。allo-HSCT至诊断IFR的中位时间为68（9～880）d。面部疼痛是最常见的临床症状（10例），其他临床症状包括鼻塞（5例）、面部肿胀（3例）、发热（2例）、头痛（1例）和失明（1例）。

5. IFR患者的诊断：13例确诊患者中，7例经组织活检+培养确诊，3例经培养确诊，3例经组织活检确诊。19例患者中，13例CT检查鼻窦有异常征象，6例MRI检查鼻窦有异常征象，16例G试验阳性，12例GM试验阳性。

6. 病原菌分布：19例IFR患者中接合菌6例（毛霉菌2例，根霉菌4例），曲霉菌4例，念珠菌3株。

7. IFR的治疗及结局：19例患者均接受抗真菌治疗，5例接受两性霉素B+泊沙康唑联合治疗，1例接受伏立康唑序贯泊沙康唑治疗，9例接受伏立康唑单药治疗，4例接受两性霉素B单药治疗。

除抗真菌药物治疗外，10例（52.6％）患者接受鼻镜下鼻内病变组织切除术+鼻窦病变手术清除术，7例获得CR，3例最终死于IFR。其余9例只接受抗真菌药物治疗，6例获得CR，2例PR。总体15例（78.9％）患者获得疗效，其中CR 13例（68.4％），PR 2例（10.5％），4例死于IFR，中位死亡时间为确诊后38（34～48）d。

8. allo-HSCT后发生IFR的危险因素：单因素分析显示患者年龄、移植前ANC，EBV感染和移植相关并发症≥3个是allo-HSCT后发生IFR的危险因素。多因素分析显示，移植前ANC<1×10^9^/L（*OR*＝22.525，*P*＝0.021）、移植后发生出血性膀胱炎（*OR*＝0.032，*P*＝0.012）、血小板延迟植入（*OR*＝1.044，*P*＝0.008）、移植物MNC（*OR*＝0.600，*P*＝0.012）是allo-HSCT后发生IFR的独立危险因素（详见表2）。

**表2:** 血液病患者allo-HSCT后发生侵袭性真菌鼻窦炎（IFR）的影响因素

变量	IFR组（19例）	对照组（95例）	单因素分析*P*值	多因素分析	
				*P*值	*OR*（95%*CI*）
年龄［例（%）］			0.245	0.579	
≤18岁	7（36.84）	49（51.58）			
>18岁	12（63.15）	46（48.42）			
性别［例（%）］			0.675	0.475	
男	10（52.6）	45（47.4）			
女	9（47.4）	50（52.6）			
移植前WBC［例（%）］			0.060	0.746	
≥4×10^9^/L	6（31.6）	53（55.8）			
<4×10^9^/L	13（68.4）	42（44.2）			
移植前ANC［例（%）］			<0.001	0.021	22.425（1.599~314.522）
≥1×10^9^/L	13（68.4）	85（89.5）			
<1×10^9^/L	6（31.6）	10（10.5）			
血小板植活时间［d，*M*（范围）］	17（9~160）	13（8~38）	0.016	0.008	1.044（1.011~1.078）
MNC输注量［×10^8^/kg，*M*（范围）］	7.47（2.54~16.00）	8.83（2.05~14.40）	0.114	0.012	0.600（0.403~0.895）
预处理方案［例（%）］			0.875	0.430	
改良Bu/Cy	3（15.8）	16（16.84）			
改良Bu/Cy+ATG	14（73.7）	72（75.79）			
Bu/Flu/Cy+ATG	0	5（5.26）			
Cy+ATG	2（10.5）	2（2.11）			
EBV感染［例（%）］	4（21.1）	9（9.5）	0.158	0.268	
出血性膀胱炎［例（%）］	3（15.8）	27（28.4）	0.262	0.012	0.032（0.002~0.470）
移植相关并发症［例（%）］			0.016	0.255	
<3个	7（36.8）	64（67.4）			
≥3个	12（63.2）	31（32.6）			

注 allo-HSCT：异基因造血干细胞移植；MNC：单个核细胞；EBV：EB病毒；Bu：白消安；Cy：环磷酰胺；Flu：氟达拉滨；ATG：抗胸腺细胞球蛋白；ANC：中性粒细胞计数

9. IFR患者的生存分析：随访至2023年9月1日，存活患者的中位随访时间为57.3（23.9～118.9）月。移植后发生IFR的19例患者中12例死亡，对照组95例患者中12例死亡，IFR组、对照组中位OS期分别为（49.90±7.07）、（108.60±3.41）月（*P*<0.01）。IFR组、对照组移植后5年OS率分别为（29.00±0.12）％、（91.00±0.03）％（*P*<0.01）。生存曲线见图1。

**图1:**
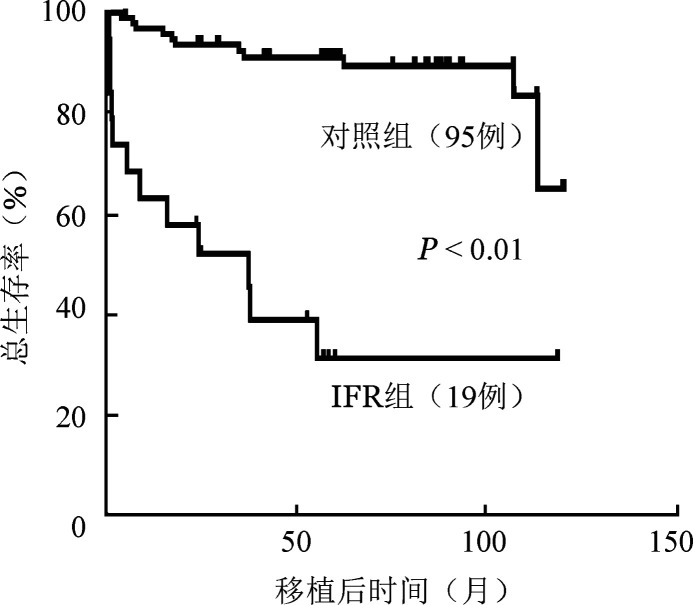
allo-HSCT后合并侵袭性真菌性鼻窦炎（IFR）和对照组的移植后总生存曲线

## 讨论

allo-HSCT后IFR相对罕见。既往文献报道，同胞全相合移植后IFR的发生率为2.6％，本研究中allo-HSCT后IFR发生率为0.22％，低于既往报道的发生率，可能因为本研究入选的均为确诊或临床诊断患者，部分疑似患者由于缺乏微生物学证据未纳入本研究。

IFR早期通常表现为鼻塞、流涕、持续性发热等非典型症状，容易漏诊或误诊。当出现面部肿胀、疼痛或麻木，以及头痛、眼球突出、癫痫等表现，则提示疾病进展以及预后不良[12]。本研究中在19例allo-HSCT后合并IFR患者的临床表现与非allo-HSCT患者类似，但进展较迅速，诊断时已经出现进展表现，其中面部疼痛是最常见的临床症状，其次为鼻塞、面部肿胀和发热。allo-HSCT患者出现面部麻木、疼痛和鼻塞时，须引起足够重视，及早进行影像学和鼻镜检查进一步明确诊断。IFR早期CT通常表现为不对称的黏膜增厚，在疾病晚期可表现为局部浸润和骨侵蚀以及眼眶、颅内受累表现。在本研究中，所有患者均出现鼻窦CT/MRI异常表现，部分患者影像学提示真菌感染可能。进一步的鼻镜检查通常可发现受累区域的结痂、黏膜苍白或坏死[13]。将临床症状、影像学与鼻镜结合有利于快速诊断。当患者临床症状及影像学考虑IFR可能时，行鼻内镜检查并进行病理活检和组织培养，除了可以确诊IFR，还可为治疗提供明确指导[14]。本研究中，84.2％（16/19）的患者都进行了鼻内镜检查，其中13例患者通过鼻窦组织培养或病理获得病原学证据。本组IFR病例中，接合菌是最常见的病原体（31.2％），其次为曲霉菌，这与其他部位侵袭性真菌感染以曲霉菌为主要病原体[15]–[16]不同。

早期诊断和立即治疗，包括抗真菌药物治疗和手术清创，对于提高IFR生存率至关重要[17]。诊断IFR后应尽早开始全身抗真菌治疗。本研究中，allo-HSCT后IFR患者长期生存率显著下降，这可能是因为本研究中患者病原体以接合菌为主，预后极差。并应根据病原学证据合理选择抗真菌药物，以改善患者预后[18]–[19]。本研究中，最常见的病原菌为接合菌，且因患者病情进展迅速，因此两性霉素B是最常用的治疗选择，部分患者接受两性霉素联合泊沙康唑治疗。除全身抗真菌治疗外，手术治疗是治疗IFR取得疗效的关键因素。在早期进行鼻内镜手术不仅可以获取组织样本明确诊断还可以对坏死组织（黏膜或骨骼）进行清除。本组19例IFR患者中，10例（83.3％）进行手术，最终7例（70％）获得CR，提示全身抗真菌药物联合手术治疗可作为allo-HSCT后IFR患者的首选治疗选择。

本组病例中，移植前患者中性粒细胞减少、移植输注MNC数量少、移植后血小板植入延迟、发生出血性膀胱炎是allo-HSCT后发生IFR的独立危险因素。这些危险因素均与患者免疫力下降密切相关，allo-HSCT后有相应危险因素的患者一旦出现鼻窦感染症状，应警惕IFR可能。

综上，本研究结果显示allo-HSCT后IFR发生率低，但预后极差。通过对IFR危险因素及早期临床表现的认识，有助于实现对allo-HSCT后IFR的早期识别和干预，全身抗真菌药物联合手术治疗可获得较好的临床疗效。

## References

[1] Sun YQ, Liu ZY, Huang XJ (2017). A retrospective study of central nervous system invasive fungal disease after allogeneic stem cell transplantation: risk factors, clinical characteristics, and outcomes[J]. Biol Blood Marrow Transplant.

[2] Gao L, Sun Y, Meng F (2016). Antifungal prophylaxis of patients undergoing allogenetic hematopoietic stem cell transplantation in China: a multicenter prospective observational study[J]. J Hematol Oncol.

[3] Deutsch PG, Whittaker J, Prasad S (2019). Invasive and non-invasive fungal rhinosinusitis-a review and update of the evidence[J]. Medicina (Kaunas).

[4] Cho HJ, Jang MS, Hong SD (2015). Prognostic factors for survival in patients with acute invasive fungal rhinosinusitis[J]. Am J Rhinol Allergy.

[5] 中国侵袭性真菌感染工作组 (2017). 血液病/恶性肿瘤患者侵袭性真菌病的诊断标准与治疗原则(第五次修订版)[J]. 中华内科杂志.

[6] Fu H, Xu L, Liu D (2014). Total body irradiation and cyclophosphamide plus antithymocyte globulin regimen is well tolerated and promotes stable engraftment as a preparative regimen before T cell-replete haploidentical transplantation for acute leukemia[J]. Biol Blood Marrow Transplant.

[7] Wang Y, Liu QF, Xu LP (2015). Haploidentical vs identical-sibling transplant for AML in remission: a multicenter, prospective study[J]. Blood.

[8] Yan CH, Wang Y, Mo XD (2018). Incidence, risk factors, microbiology and outcomes of pre-engraftment bloodstream infection after haploidentical hematopoietic stem cell transplantation and comparison with hla-identical sibling transplantation[J]. Clin Infect Dis.

[9] Huang XJ, Liu DH, Liu KY (2006). Haploidentical hematopoietic stem cell transplantation without in vitro T-cell depletion for the treatment of hematological malignancies[J]. Bone Marrow Transplant.

[10] Huang XJ, Liu DH, Liu KY (2009). Treatment of acute leukemia with unmanipulated HLA-mismatched/haploidentical blood and bone marrow transplantation[J]. Biol Blood Marrow Transplant.

[11] Harris AC, Young R, Devine S (2016). Multicenter standardization of acute graft-versus-host disease clinical data collection: a report from the Mount Sinai Acute GVHD International Consortium[J]. Biol Blood Marrow Transplant.

[12] Melancon CC, Clinger JD (2017). The use of frozen section in the early diagnosis of acute invasive fungal sinusitis[J]. Otolaryngol Head Neck Surg.

[13] Thompson GR, Patterson TF (2012). Fungal disease of the nose and paranasal sinuses[J]. J Allergy Clin Immunol.

[14] Shamsaei S, Falahati M, Farahyar S (2021). Acute invasive fungal rhinosinusitis: Molecular identification and update in management of frozen section biopsy[J]. Microb Pathog.

[15] Kim DH, Kim SW, Hwang SH (2021). Usefulness of intraoperative frozen section for diagnosing acute invasive fungal rhinosinusitis: A systematic review and meta-analysis[J]. Int Forum Allergy Rhinol.

[16] Pinto TA, Jardim BA, Breda GL (2020). Infectious complications in pediatric allogeneic hematopoietic stem cell transplantation recipients-A retrospective clinical and epidemiological cohort study[J]. Transpl Infect Dis.

[17] Chakrabarti A, Denning DW, Ferguson BJ (2009). Fungal rhinosinusitis: a categorization and definitional schema addressing current controversies[J]. Laryngoscope.

[18] Tissot F, Agrawal S, Pagano L (2017). ECIL-6 guidelines for the treatment of invasive candidiasis, aspergillosis and mucormycosis in leukemia and hematopoietic stem cell transplant patients[J]. Haematologica.

[19] Kably B, Launay M, Derobertmasure A (2022). Antifungal drugs TDM: trends and update[J]. Ther Drug Monit.

